# A positive Alzheimer’s blood biomarker is not a diagnosis: the presence–primacy–permission framework for plasma p-tau217

**DOI:** 10.1097/MS9.0000000000005661

**Published:** 2026-09-08

**Authors:** Vishal Bhatia, Adnan Khatak

**Affiliations:** a Department of Internal Medicine, Ghulam Muhammad Mahar Medical College, Sukkur, Pakistan; b Department of Internal Medicine, Nangarhar University, Jalalabad, Afghanistan

**Keywords:** Alzheimer disease, anti-amyloid therapy, blood-based biomarker, diagnostic error, phosphorylated tau 217

## Abstract

Validated plasma phosphorylated tau 217 (p-tau217) assays identify Alzheimer disease pathology with high accuracy. Wider clinical use creates a risk of postanalytic misclassification when pathology is converted directly into a clinical diagnosis or treatment decision. This editorial examines why accuracy relative to amyloid positron emission tomography or cerebrospinal fluid biomarkers does not establish that Alzheimer disease is the principal cause of a patient’s cognitive syndrome. It describes three substitutions: pathology for diagnosis, presence for primacy, and diagnosis for treatment eligibility. We propose the presence–primacy–permission framework to distinguish confirmation of pathology from clinical-biological concordance and treatment suitability. Presence requires appropriate patient selection, a validated assay, and context-specific interpretation. Primacy requires integration of cognitive phenotype, trajectory, functional status, neurologic examination, structural imaging, and coexisting pathology. Permission requires assessment of clinical stage, hemorrhagic risk, treatment feasibility, and patient goals. Separate documentation of the biomarker result, syndrome, etiologic attribution, and actionability may reduce premature diagnostic closure while preserving the clinical value of p-tau217.

## Introduction

Consider an older adult referred for progressive cognitive change. Neuropsychological testing shows executive dysfunction, neurologic examination reveals gait slowing and subtle parkinsonism, and magnetic resonance imaging (MRI) shows substantial small-vessel ischemic disease. Plasma phosphorylated tau 217 (p-tau217) is positive. The record lists “Alzheimer’s disease,” subsequent care becomes organized around that label, and referral for anti-amyloid therapy follows. Yet the biomarker result and the clinical conclusion do not answer the same question.

Validated plasma p-tau217 assays identify Alzheimer disease pathology with high accuracy[1]. However, agreement with amyloid positron emission tomography (PET) or cerebrospinal fluid (CSF) biomarkers does not establish that the detected pathology is the principal cause of the patient’s cognitive impairment[1]. Older adults commonly have multiple cerebral pathologies. Depending on phenotype, disease stage, imaging findings, and competing explanations, a positive result may indicate Alzheimer disease as the dominant cause, a contributing process, or a finding of uncertain clinical relevance[2].

As access to testing expands, the risk is not limited to analytic false-positive results. A technically valid result can still produce postanalytic misclassification when pathology is converted directly into an etiologic diagnosis or a treatment decision. Such premature closure may obscure treatable contributors, fail to recognize mixed disease, distort prognostic counseling, and prompt inappropriate treatment pathways. This editorial proposes a structured approach based on three judgments: pathological presence, etiologic primacy, and therapeutic permission.

## Why high accuracy does not settle causation

Diagnostic performance is evaluated against amyloid PET or CSF evidence of Alzheimer disease pathology. The area under the receiver operating characteristic curve (AUC) describes how well an assay discriminates between pathology-positive and pathology-negative individuals. Sensitivity and specificity describe this classification at a selected cutoff. None of these measures determines whether the detected pathology explains the patient’s symptoms.

Predictive values add another layer of context. Positive predictive value (PPV) depends on the prevalence of Alzheimer’s disease pathology in the tested population and, therefore, on the patient’s pretest probability. The same result may carry different implications in a specialist memory clinic, primary care, or an unselected population. In a 2025 multicohort study of 1767 symptomatic patients, the fully automated Lumipulse p-tau217 assay achieved AUCs of 0.93 to 0.96. In primary care, a single cutoff produced 85% accuracy and a PPV of 82%. A two-cutoff strategy increased accuracy to 92% and classified 16% of patients as intermediate[3]. This gray zone reflects clinically appropriate uncertainty, not test failure.

Plasma p-tau217 should not be treated as a uniform test. Absolute p-tau217 concentrations, p-tau217-to-amyloid-β42 ratios, and the percentage of p-tau217 measured by immunoassay or mass spectrometry have different cutoffs and performance characteristics. The Alzheimer’s Association recommends selecting and interpreting blood-based biomarkers according to assay-specific performance and within a comprehensive clinical evaluation[4]. For the patient in the vignette, a positive result may reliably indicate Alzheimer disease pathology, while leaving unresolved whether vascular injury, Lewy body disease, Alzheimer disease, or their combination best explains the executive-predominant syndrome.

## The three diagnostic substitutions

The first substitution converts pathology into a diagnosis. Once a positive result appears in the record, “Alzheimer pathology detected” may become “Alzheimer disease” without a documented cognitive syndrome. This removes the distinction between a biological finding and a clinical disorder. The US Food and Drug Administration makes this distinction explicit: the cleared plasma p-tau217/amyloid-β42 ratio is an aid for evaluating symptomatic patients and is not intended for screening or as a stand-alone diagnostic test[5]. In the vignette, objective impairment is present, but the executive-predominant profile, gait disorder, and subtle parkinsonism require explanation rather than being subsumed under a biomarker label.

The second substitution converts presence into primacy. Alzheimer disease pathology may be the dominant cause, a contributor to another disorder, or insufficient to explain the severity and phenotype of impairment. Older adults frequently harbor vascular lesions, α-synuclein, transactive response DNA-binding protein 43 (TDP-43), and other coexisting pathologies alongside amyloid and tau[6]. A positive p-tau217 result should increase confidence that Alzheimer disease biology is present, but it should not reduce attention to discordant features. For this patient, the tempo of decline, informant history, neuropsychological pattern, motor findings, vascular burden, and features of Lewy body disease remain clinically decisive. The appropriate conclusion may be Alzheimer disease, cognitive impairment due to mixed etiologies, or a syndrome for which etiologic attribution remains uncertain.

The third substitution converts diagnosis into treatment eligibility. Confirmation of amyloid pathology is required before anti-amyloid therapy, but it does not establish eligibility. Lecanemab and donanemab were studied in early symptomatic disease, and appropriate-use recommendations require a compatible clinical stage and product-specific safety assessment[7]. Baseline MRI findings, cerebral microhemorrhages, superficial siderosis, possible cerebral amyloid angiopathy, anticoagulant use, apolipoprotein E (APOE) genotype, monitoring capacity, comorbidities, and patient goals can materially alter the benefit-risk judgment. In the vignette, vascular imaging abnormalities that support an alternative or additional diagnosis may also affect treatment safety. Referral may be reasonable, but it should request an eligibility assessment rather than imply eligibility. Separating pathology, etiologic attribution, and treatment suitability preserves the value of p-tau217 without granting the test more diagnostic authority than its evidence supports.

## The presence–primacy–permission framework

The framework separates three questions that are often compressed into one clinical label.

### Presence: Is Alzheimer disease pathology present?

Testing should begin in an appropriate clinical population. Current specialty-care guidance supports blood-based biomarker testing after a comprehensive assessment has established objective cognitive impairment, rather than for population screening or subjective concern alone^[^4,5^]^. The clinician should estimate pretest probability from age, syndrome, disease stage, and setting, and then select a validated assay whose intended use and performance match the clinical purpose. Cutoffs are not interchangeable across absolute p-tau217, percentage p-tau217, and p-tau217/amyloid-β42 assays. Renal impairment may increase absolute p-tau217 concentrations and the proportion of intermediate results, although its effect on diagnostic discrimination varies by assay and study[3]. Kidney function should therefore inform interpretation rather than automatically invalidate a result. A value within the indeterminate zone should remain indeterminate. If establishing pathology would alter etiologic attribution or treatment eligibility, amyloid PET or CSF testing may be appropriate rather than forcing a binary conclusion.

### Primacy: Is Alzheimer’s disease pathology the principal cause of the syndrome?

This gate begins with the clinical trajectory. Clinicians should define onset, tempo, fluctuation, and the earliest affected cognitive domains using accounts from both the patient and a knowledgeable informant. Standardized cognitive testing should establish the cognitive pattern, while functional history distinguishes mild cognitive impairment from dementia and identifies discrepancies between test performance and everyday ability. Neurologic examination should assess parkinsonism, gait dysfunction, focal deficits, eye movement abnormalities, motor neuron signs, and other phenotype-defining findings. MRI is not merely an exclusion test. The distribution and severity of atrophy, infarction, white matter disease, microhemorrhages, and hydrocephalus may support, modify, or challenge etiologic attribution. Alzheimer disease pathology can be dominant, contributory, or coincident with vascular, Lewy body, TDP-43-related, or other pathology[6]. When the phenotype and biomarker results are discordant, further evaluation should address the discordance rather than allowing a positive result to prematurely finalize the diagnosis.

### Permission: Is disease-modifying treatment appropriate for this patient?

Treatment assessment requires more than biomarker positivity. The patient should have a compatible clinical stage and a reasonable likelihood that Alzheimer’s disease is driving the current impairment. Baseline MRI should assess microhemorrhages, superficial siderosis, prior macrohemorrhage, extensive vascular injury, and other indicators of hemorrhagic risk and amyloid-related imaging abnormalities (ARIA). APOE genotyping, with counseling and consent when relevant, can refine risk discussions. Anticoagulant or antiplatelet use, the potential need for thrombolysis, unstable comorbidity, and interacting therapies require product-specific review[7]. The clinical service must be able to provide scheduled imaging, treatment monitoring, urgent assessment of neurologic symptoms, and management of ARIA. Finally, expected benefit, residual uncertainty, treatment burden, cost, access, and patient goals should be discussed with the patient and care partner.

## Conclusion

Plasma p-tau217 represents an important advance because validated assays can identify Alzheimer’s disease pathology more accessibly than CSF testing or PET imaging. Its value depends on disciplined interpretation. The central concern is not only whether a p-tau217 assay is sufficiently accurate, but whether clinicians ask it to answer questions it was not designed to answer. A positive result supports the presence of pathology; it does not independently establish etiologic primacy or confer therapeutic permission. Preserving these distinctions may prevent premature closure, maintain attention to reversible contributors and coexisting pathology, and improve prognostic and treatment discussions. The appropriate response to a positive biomarker result is neither dismissal nor automatic diagnostic conversion. It is a structured clinical synthesis that states what the test establishes, what remains uncertain, and what additional evidence is required before making a diagnosis or initiating treatment.

## Ethical approval

Not applicable. This editorial is based exclusively on previously published literature and did not involve human participants, patient-level data, or animal subjects.

## Consent

Not applicable. No patient information or identifiable patient data are included in this editorial.

## Data Availability

No new data were generated or analyzed for this editorial. Data sharing is therefore not applicable.
